# Diagnostic Value of Clinical Findings in Evaluation of Thoracolumbar Blunt Traumas

**Published:** 2016

**Authors:** Ali Shahrami, Majid Shojaee, Seyed Mohammadreza Tabatabaee, Elaheh Mianehsaz

**Affiliations:** 1 Emergency Department, Imam Hossein Hospital, Shahid Beheshti University of Medical Sciences, Tehran, Iran.; 2 Clinical Research Support Center, Kashan University of Medical Sciences, Kashan, Iran.

**Keywords:** Spinal fractures, Physical examination, Tomography, X-ray computed, Signs and symptoms

## Abstract

**Introduction:**

Necessity of imaging for symptom-free conscious patients presented to emergency department (ED) following traumatic thoracolumbar spine injuries has been a matter of debate. The present study was aimed to evaluate the diagnostic value of clinical findings in prediction of traumatic thoracolumbar injuries compared tocomputed tomography (CT) scan.

**Methods:**

The present diagnostic value study was carried out using non-random convenience sampling during the time between October 2013 and March 2014. All trauma patients > 15 years old underwent thoracolumbar CT scan were included. Correlation between clinical and CT findings was measured using SPSS 21.0 and screening performance characteristics of clinical findings in prediction of thoracolumbar fracture were calculated.

**Results:**

169 patients with mean age of 37.8 ± 17.3 years (rage: 15-86) were evaluated (69.8% male). All fracture patients had at least 1 positive finding in history and physical examination. The fracture was confirmed in only 24.6% of the patients with positive findings in history or physical examination. In 37.5% of patients the location of fracture, matched the area of positive physical examinations. Sensitivity, specificity, PPV, NPV, PLR, and NLR of clinical findings in comparison to thoracolumbar CT scan were 100 (95% CI: 89 - 100), 1.5 (95% CI: 0.2-6), 24.5 (95% CI: 18.3-31.9), 100 (95% CI: 19.7-100), 32.5 (95% CI: 24.6-43.03), and infinite, respectively.

**Conclusion:**

The results of the present study, show the excellent screening performance characteristics of clinical findings in prediction of traumatic thoracolumbar fracture (100% sensitivity). It could be concluded that in conscious patients with stable hemodynamic, who have no distracting pain and are not intoxicated, probability of thoracolumbar fracture is very low and near to zero in case of no positive clinical finding.

## Introduction:

Multiple trauma patients make up a considerable portion of emergency department (ED) visitors. Spine is prone to injury following trauma. Traumatic spinal injuries bring about various and sometimes dangerous outcomes such as spinal cord injury, neurologic injuries, chronic pain, deformity, transient or permanent functional disorders, and decreased quality of life (1-3). About 50% of spinal fractures occur in the thoracolumbar area and 19% to 50% of them lead to neurologic injuries (4, 5). Incidence of traumatic spinal cord injuries has been reported to be 3.6 cases per million population in Canada and 23 per million around the world. In Iran, due to various reasons such as high rate of traffic accidents and not conducting occupational safety, the incidence has been much higher and estimated to be about 30 to 72.4 cases per million (6). Misdiagnosis incidence has been reported to be up to 20% in thoracolumbar blunt traumas. Late diagnosis of these injuries leads to 7-8 times increase in neurologic complications (4, 5, 7, 8). Therefore, early diagnosis and triage of the patients in need of complimentary diagnostic and curative measures is of great importance in managing these patients. Most researchers believe thoracolumbar trauma patients with pain, tenderness, positive neurologic findings, loss of consciousness, multiple trauma, and those unable to go through detailed physical examination should undergo imaging. (4, 9-11) Necessity of imaging for symptom-free conscious patients has been a matter of debate (10, 12-19). Using clinical decision rules such as National Emergency X-Radiography Utilization Study (NEXUS) and Canadian C-spine rule can be helpful in this regard (20-22). Despite the higher prevalence of thoracolumbar area injuries compared to cervical ones, less research has been done on trauma in this area and there is much controversy regarding its management (9, 23). Although physical examination is highly important in diagnosis of thoracolumbar area injuries, its accuracy is a matter of question. Based on a review study, physical examination can only predict 27-56% of thoracolumbar fractures in patients with blunt trauma and its sensitivity and specificity have been estimated to be 48.2% and 84.9%, respectively (4). Based on the afore-mentioned points, the present study was aimed to evaluate the diagnostic value of clinical findings in prediction of thoracolumbar traumatic spinal injuries in comparison with computed tomography (CT) scan.

## Methods:

***Study design and setting***

The present diagnostic value study was carried out using non-random convenience sampling during the time between October 2013 and March 2014 in the ED of Imam Hossein Hospital, Tehran, Iran.

***Inclusion and exclusion criteria***

All patients over 15 years of age who were presented to the ED following multiple trauma and underwent thoracolumbar CT scan were included. Patients with decreased level of consciousness, hemodynamic instability, distracting pain, intoxicated following alcohol, opiate or sedative drug use, and those who did not fully participate during physical examination for any reason were excluded.

In the present study, positive clinical finding was considered the presence of any of the following items in history of physical examination: back pain, midline tenderness, lateral tenderness, skin abrasion or laceration, and focal neurologic deficit (sensory/motor). 

***Data gathering***

Demographic data (age, sex), trauma mechanism (traffic accident, falling, assault, direct collision with a h object, rubble), history and physical examination findings (thoracolumbar pain, midline or lateral thoracolumbar tenderness, bruises and laceration, step or gap touch, deformity, neurological sensorimotor disorder) and thoracolumbar CT scan findings regarding presence or absence of fracture(s) were gathered and recorded using a checklist. History was obtained and physical examination was performed by a third-year emergency medicine resident. All CT scans were interpreted by an independent radiologist blind to the clinical findings. This study did not interfere with the treatment process and imaging performance in the patients. 

***Statistical analyses***

SPSS version 21 was used for statistical analyses. The required sample size for this study was estimated to be 160 cases, based on 48.2% sensitivity of the clinical findings compared to CT scan in vertebra fracture (16), α = 0.1, Z_1-α/2 _= 1.96, L = 0.1, and S_N_ = 0.482. The results were reported as mean ± standard deviation (SD) for quantitative data and as frequency and percentage for qualitative ones. Quantitative data were compared using t-test and Mann-Whitney test and qualitative ones were compared using Chi-square and Fisher’s exact tests. Correlation between quantitative variables was measured using Pearson correlation coefficient and Spearman rank correlation. Sensitivity, specificity, positive and negative predictive values (PPV and NPV), and positive and negative likelihood ratios (PLR and NLR) of clinical findings in prediction of thoracolumbar fracture were determined. Thoracolumbar CT scan was considered as the standard reference test. p < 0.05 was defined as significance level.

**Table 1:** Baseline characteristics of the participants

**Variables **	**Number of patients (%)**	
	**Without fracture**	**With fracture**
**Age (years)**		
< 20	21 (12.4)	3 (7.5)
20-30	55 (32.5)	13 (32.5)
30-40	30 (17.7)	5 (12.5)
40-50	23 (13.6)	8 (20)
50-60	19 (11.2)	6 (15)
> 60	21 (12.4)	5 (12.5)
**Fracture site**		
Thoracic	17 (10)	17 (42.5)
Lumbar	22 (13)	22 (55)
Thoracolumbar	1 (0.6)	1 (2.5)
**Trauma mechanism**		
Collision	93 (55)	20 (50)
Falling down	49 (29)	14 (35)
Assault	7 (1.4)	1 (2.5)
Hard object collision	18 (10.7)	5 (12.5)
Rubble	2 (1.1)	0 (0)
**Fracture type**		
Compression or wedge	15 (9)	15 (37.5)
Burst	6 (4)	6 (15)
Chance	1 (0.6)	1 (2.5)
Fracture-dislocation	2 (1.1)	2 (5)
Body and process	4 (2.3)	4 (10)
Transverse process	9 (5.3)	9 (22.5)
Spinous process	2 (1.1)	2 (5)
Facet or articular process	0 (0)	0 (0)
Pars inter-articular or listhesis	1 (0.6)	1 (2.5)

**Table 2:** History and physical examination of the patients

**Findings**			**Number of patients (%)**	
			**Without fracture**	**With fracture**
**Back pain**	No	8 (4.7)		3 (1.8)
	Yes	121 (71.6)		37 (21.9)
**Midline tenderness**	No	101 (59.8)		10 (5.9)
	Yes	28 (16.6)		30 (17.8)
**Lateral tenderness**	No	90 (53.3)		19 (11.2)
	Yes	39 (23.1)		21 (12.4)
**Abrasion**	No	65 (38.5)		18 (10.7)
	Yes	64 (37.9)		22 (13)
**Neurologic examination**	Normal	128 (75.7)		37 (21.9)
	Abnormal	1 (0.6)		3 (1.8)
**Movement**	Not moving	2 (1.2)		3 (1.8)
	Just lateral	4 (2.4)		5 (3)
	Can sit	11 (6.5)		10 (5.9)
	Can stand up	5 (3)		5 (3)
	Can walk	107 (63.3)		17 (10.1)

## Results:

 169 patients with mean age of 37.8 ± 17.3 years (rage: 15-86) were evaluated (69.8% male). Table 1 shows baseline characteristics of the studied patients. The most frequent fractures are second and third lumbar vertebrae fractures with 11 (27.5%) and 8 (20%) cases, respectively, and 12th thoracic vertebra with (20%). Table 2 summarizes the findings of patients’ history and physical examination. All fracture patients had at least 1 positive finding in history and physical examination. The fracture was confirmed in only 24.6% of the patients with positive findings in physical examination or history. In 37.5% of patients the location of fracture, matched the area of positive physical examinations, while in 40% of the cases, the area affected with tenderness was bigger than the fractured vertebra. Sensitivity, specificity, PPV, NPV, PLR, and NLR of clinical findings in comparison to thoracolumbar CT scan were 100 (95% CI: 89 - 100), 1.5 (95% CI: 0.2-6), 24.5 (95% CI: 18.3-31.9), 100 (95% CI: 19.7-100), 32.5 (95% CI: 24.6-43.03), and infinite, respectively.

## Discussion:

Based on the results of this study, history and physical examination have 100% sensitivity and NPV in screening thoracolumbar trauma patients. In fact, the probability of thoracolumbar fracture in conscious patients with stable hemodynamic, who have no positive finding in history and physical examination, is very low. In the present study, like previous ones, men were affected by trauma and thoracolumbar spinal fracture more than women, and the 20-30 years age range had the highest prevalence of fracture (4, 24-26).

In this study, the most frequent trauma mechanism was traffic accidents, especially pedestrian-car accidents, which was in line with the results of Yousefzadeh and Inaba (17, 24). In contrast, studies by Heidary (25), Rahimi-Movaghar (27), and Fakharian (26) in Iran, and Karamehmetoglu in Turkey (28) reported falling as the most prevalent mechanism for thoracolumbar spinal trauma. In most studies, as well as the present one, the most common type of fracture was compression (4, 16). While, Fakharian et al. showed that burst fracture was more common. They believed that the studied population being neurosurgery patients was the reason for this difference (26). 

In the present study, no difference was detected regarding clinical findings neither between men and women nor in various age ranges. Back pain was the most common complaint in patients with or without spinal fracture. Also in patients with spinal fracture, midline tenderness was the most common symptoms. The prevalence of positive neurologic findings in physical examination was 2.4%. In contrast, this rate was estimated to be 18% in Yousefzadeh et al. study, 17% in the Fakharian et al. study, and 20% in Hsu JM et al. study (9, 24, 26). The reason for the low rate of positive neurologic findings in the present study can be excluding patients with distracting pain, decreased level of consciousness, and intoxicated; as logically the prevalence of neurologic disorders may be higher in this group due to more severe trauma.

In a study by Frankel et al. 60% and in the one by Cooper et al. 31% of the patients with thoracolumbar fracture were symptom-free (29, 30). In our study, 15% of these patients had normal physical examinations.

In 37.5% of patients with fracture, the area of positive physical examinations was completely in agreement with the true site of injury in CT scan, and in 40% of the patients, the area affected with tenderness was bigger than the fractured area in the CT scan. In the Inaba et al. study, agreement rate between the site of pain and fracture was 61.6% (17). 

Sensitivity of physical examination in predicting traumatic thoracolumbar spine injuries has been estimated to be about 48-85% in various studies (17, 31). The results of the present study show 100% sensitivity of history and physical examination in ruling out thoracolumbar fractures. Samuel et al. also showed that normal history and physical examination are enough to rule out the probability of fracture (16). In short, clinical findings seem to be valuable in management of patients presented to the ED following traumatic thoracolumbar injuries.

**Limitations and suggestions**

Since patients with distracting injuries (patients with abdominal or chest problems or big bone fracture) were excluded, calculation of injury severity score had no value. It is suggested to carry out a more comprehensive study without excluding these patients and calculate sensitivity and specificity of various items of physical examination in all thoracolumbar trauma patients including conscious and unconscious ones, with and without distracting injuries. Short and long term follow-up of the patients, which are routinely managed based on clinical findings and discharged without undergoing imaging study, can be very helpful in confirmation and external validation of this management approach.

## Conclusion:

The results of the present study, show the excellent screening performance characteristics of clinical findings in prediction of traumatic thoracolumbar fracture (100% sensitivity). It could be concluded that in conscious patients with stable hemodynamic, who have no distracting pain and are not intoxicated, probability of thoracolumbar fracture is very low and near to zero in case of no positive clinical finding.
